# On Modern Progress in Ophthalmic Medicine and Surgery

**Published:** 1895-06-22

**Authors:** Robert Brudenell Carter

**Affiliations:** Consulting Ophthalmic Surgeon to St. George's Hospital


### On Modern Progress in Ophthalmic Medicine and Surgery.

By Robert Brudenell Carter, F.R.C.S., Consulting Ophthalmic Surgeon to St. George's Hospital.

### DISEASES OF THE CORNEA.

*(Continued from page 125.)*

The next most important form of keratitis, the so-called "interstitial," has long been recognised as furnishing a distinct type of disease, and is to be found described in old text-books as one of the many forms of what was called "strumous ophthalmia." It was probably far more common five-and-thirty years ago than it is now; and Mr. Hutchinson, taking notes of the cases that presented themselves at Moorfields, and inquiring into their family histories, was struck by the frequency with which the mothers of the patients had been the subjects of miscarriage, and with the prevalence, among the patients themselves, of certain deformities of the teeth and certain peculiarities of facial outline and expression. Some years earlier, the late Sir William Wilde had observed the frequent association of this form of "strumous ophthalmia" with deafness, and had discovered that it could be best treated by the administration of perchloride of mercury; but he just missed what Mr. Hutchinson established, namely, that the disease was essentially syphilitic, and that it chiefly occurred in children whose parents could be shown to have suffered from that malady. The teeth and facies described by Mr. Hutchinson were soon admitted to be generally associated with the corneal inflammation, and the whole series of morbid phenomena was regarded as a manifestation of inherited syphilis.

There were, notwithstanding, some dissentients from this view, among practitioners thoroughly acquainted with the family histories of certain patients, and confident that in these histories syphilis had no place. There seemed to be no doubt that the affection was specific in the majority of cases, but in some the source of infection could not be traced; while, on the other hand, the uniformity of the symptoms was so striking as almost to preclude the idea that the affection could be specific in some instances and not in others. A family which came under my own notice, and in which there was a single very conspicuous example of keratitis, with the associated teeth and facies, among a number of perfectly healthy brothers and sisters, and with perfectly healthy parents, gave me the impression that the disease in the isolated case might be vaccinal and not inherited; and I stated this opinion several years ago, at a time when the very possibility of vaccinal syphilis was denied by many authorities. That possibility has now been abundantly established, and has led to such a degree of care in the selection of vaccinifers that the danger has practically been entirely overcome. The very foolish persons who agitate against vaccination have over and over again represented, with the characteristic disingenuousness of faddists, that I am a supporter of their views; and, although I have repeatedly denied the imputation, I have no expectation that those who made it will be silenced. The occasional occurrence of constitutional infection through vaccination, even if it were unavoidable, would furnish no better argument for the abandonment of the practice than would the occasional occurrence of a carriage accident for the abandonment of wheeled conveyances; but, in point of fact, to be aware of the former danger is to be in a position to exclude it.

Interstitial keratitis commences with some diminution of the previous acuteness of vision, and this, on inspection of the eye, is discovered to be due to a slight nebulosity of the cornea, chiefly at its central part, a nebulosity which, in the words of Mr. Hutchinson, when examined with a magnifying glass, looks as if it were composed of microscopic masses of fog. At first, as a rule, there is no increased vascularity of the eye, but symptoms of congestion and irritation soon appear, and the eye becomes flushed, watery, and somewhat tender or painful. At the same time the nebulosity increases, and the vision as steadily diminishes. The disease tends naturally to run a prolonged course, and to attack the second eye, and the cloudiness may become so marked as practically to extinguish vision for a time, but we never see the peculiar dense opacity which follows the acute stage of vascular keratitis, and the ultimate clearing of the cornea is usually nearly complete.

In interstitial, as in vascular keratitis, the application of any irritant drops or lotion will invariably aggravate the symptoms, and may even bring about a condition in which the cornea becomes traversed by numerous vessels, and in which the disease will be both more prolonged and more injurious than in ordinary cases. The patients are generally feeble children, or, if past childhood, are persons in whom the appearance of the malady has been determined by some depressing conditions. Apart from exceptional conditions of the general health, which may require attention as they appear, mercury, as found by Sir William Wilde, must be regarded as the sheet-anchor of treatment, and may sometimes be combined with iodide of potassium and ammonio-citrate of iron. The perchloride is, I think, generally the best and most trustworthy preparation; and the method of administration by sub-conjunctival injection, which I described in a recent paper, may sometimes be adopted with advantage. Care should be taken that none of the solution is suffered to remain on the surface of the conjunctiva, in which situation it might irritate the cornea. At some period of the case there is often much intolerance of light; and any such period should be tided over by temporary confinement to a dimly-lighted room, in which, if necessary, exercise may be taken with a skipping-rope or in some other suitable manner. Functional rest of the eyes should be enforced; cocaine and atropine instilled; the diet should be simple and sufficient, and the clothing light and warm. Complications should be met as they arise; increased congestion by a leech, pain or irritation by a more strict enforcement of repose, and by local sedatives. Such complications, however, will seldom give serious trouble, unless they have been brought about by altogether unreasonable neglect of some of the precautions which prudence would obviously demand. The patients require to be nursed carefully, during the time that is necessary in order to bring the influence of mercurial treatment to bear fully upon the essential cause of the malady.

